# Supplementary material for: A high-resolution mRNA expression time course of embryonic development in zebrafish
Source: eLife. 2017 Nov 16;6:e30860. doi: 10.7554/eLife.30860 (PMC5690287; doi:10.7554/eLife.30860)
Supplement: Supplementary file 6. [file elife-30860-supp6.zip › biolayout-clusters-files/Cluster187-genes.html]

Cluster187


# Cluster187: Genes

| Ensembl ID | Gene Name | Chr | Start | End | Biotype |
| --- | --- | --- | --- | --- | --- |
| ENSDARG00000001890 | STT3B | 16 | 39435451 | 39527714 | protein\_coding |
| ENSDARG00000043658 | cxadr | 10 | 27134330 | 27196527 | protein\_coding |
| ENSDARG00000041493 | ipo4 | 24 | 12607710 | 12686009 | protein\_coding |
| ENSDARG00000075100 | neo1b | 25 | 2623429 | 2887019 | protein\_coding |
| ENSDARG00000021808 | nmt2 | 16 | 28643729 | 28656918 | protein\_coding |
| ENSDARG00000024277 | pigm | 24 | 42180468 | 42187198 | protein\_coding |
| ENSDARG00000002830 | trmt2a | 5 | 24028601 | 24039726 | protein\_coding |
